# Nuss procedure for the treatment of pectus excavatum with dyspnea following oropharyngeal cancer surgery: a case report

**DOI:** 10.1093/jscr/rjad714

**Published:** 2024-01-18

**Authors:** Kyohei Masai, Taketo Nakai, Yu Okubo, Kaoru Kaseda, Tomoyuki Hishida, Keisuke Asakura

**Affiliations:** Division of Thoracic Surgery, Department of Surgery, Keio University School of Medicine, Tokyo 160-8582, Japan; Division of Thoracic Surgery, Department of Surgery, Keio University School of Medicine, Tokyo 160-8582, Japan; Division of Thoracic Surgery, Department of Surgery, Keio University School of Medicine, Tokyo 160-8582, Japan; Division of Thoracic Surgery, Department of Surgery, Keio University School of Medicine, Tokyo 160-8582, Japan; Division of Thoracic Surgery, Department of Surgery, Keio University School of Medicine, Tokyo 160-8582, Japan; Division of Thoracic Surgery, Department of Surgery, Keio University School of Medicine, Tokyo 160-8582, Japan

**Keywords:** pectus excavatum, cardiopulmonary dysfunction, tracheal deviation, bronchial stenosis

## Abstract

Pectus excavatum (PE) causes cardiopulmonary dysfunction depending on the degree and form of the depression. The patient was a 74-year-old woman with a history of PE. Fourteen years ago, a total glossolaryngectomy was performed for oropharyngeal cancer. Two years later, the patient gradually experienced difficulty in breathing. Computed tomography (CT) revealed severe PE and right main bronchial stenosis. We performed a Nuss procedure for PE repair to surgically release the stenosis of the right main bronchus. Postoperative chest CT showed improvement in the sternal depression and right main bronchial stenosis. Furthermore, shortness of breath was relieved postoperatively. Oropharyngeal cancer surgery may cause tracheal support disruption, leading to leftward shift and severe stenosis of the right main bronchus due to sternum depression. This is an important report regarding respiratory distress caused by a combination of PE and post-oropharyngeal cancer surgery.

## Introduction

Pectus excavatum (PE) is a common chest wall deformity that may lead to cardiopulmonary dysfunctions [1, 2]. The symptoms vary depending on the degree and location of the chest depression. Changes in the thoracic cavity after other thoracic surgeries should be considered in patients with PE. We present a novel case of PE, with dyspnea following cancer surgery, treated with the Nuss procedure. The mechanism that caused airway obstruction is crucial to discuss.

## Case report

**Figure 1:**
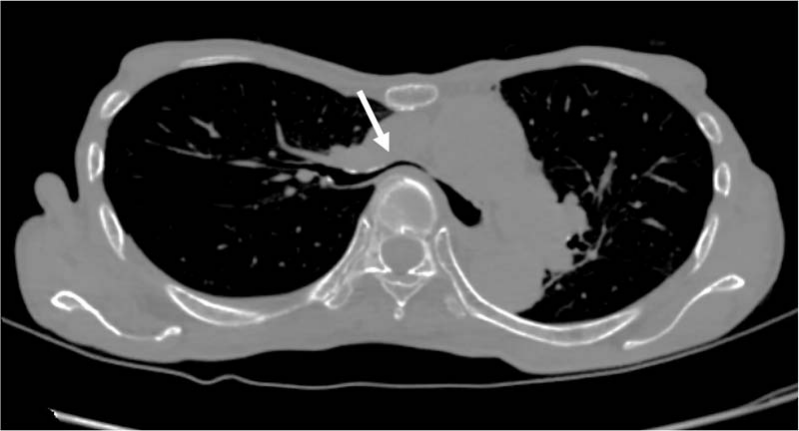
Chest CT findings of severe PE with stenosis of the right main bronchus. The right main bronchus was severely narrowed with a HI (HI) of 8.9 (arrow).

**Figure 2:**
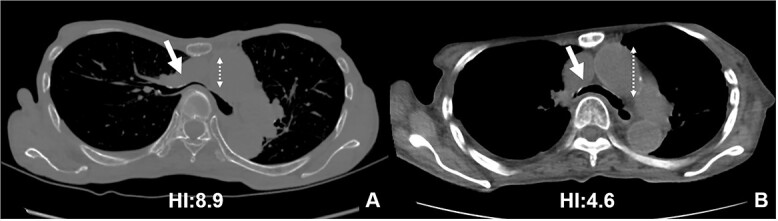
CT scans are used to monitor changes over time. In (A), chest CT findings of severe PE with stenosis of the right main bronchus. The trachea, which was centrally located on CT scan 8 years ago (2B), has shifted to the left now (2A). Additionally, the CT-HI worsened over his 8 years, increasing from 6.8 to 8.9.

The patient was a 74-year-old woman with a history of childhood PE. Fourteen years ago, a total glossolaryngectomy was performed at another hospital for oropharyngeal cancer, and a permanent tracheal stoma was created. Approximately 2 years later, the patient gradually experienced difficulty in breathing. Chest computed tomography (CT) revealed severe PE and right main bronchial stenosis. Subsequently, the patient was referred to our department. A preoperative spirometry test showed a vital capacity of 1.14 L (45% of the predicted value). As part of the imaging test, a chest CT indicated a leftward deviation of the trachea. Symmetrical depression with a Haller Index (HI) of 8.9 and stenosis of the right main bronchus were observed at the same site (Fig. 1). The chest CT taken over 8 years indicated that the leftward deviation of the trachea had progressed and the thoracic depression had worsened (Fig. 2). Intraluminal observation using a bronchoscopy revealed severe stenosis of the right main bronchus, making peripheral observation challenging (Fig. 4A).

**Figure 3:**
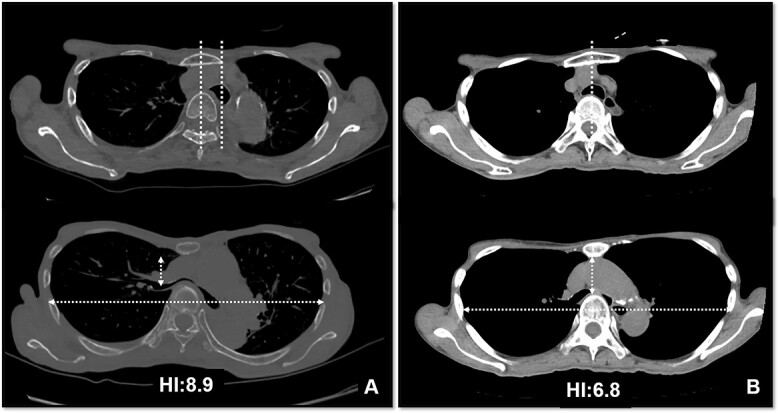
Pre- (A) and postoperative (B) CT findings: the Nuss procedure improved the chest wall and right main bronchus stenosis, and reduced HI from 8.9 to 4.6.

**Figure 4:**
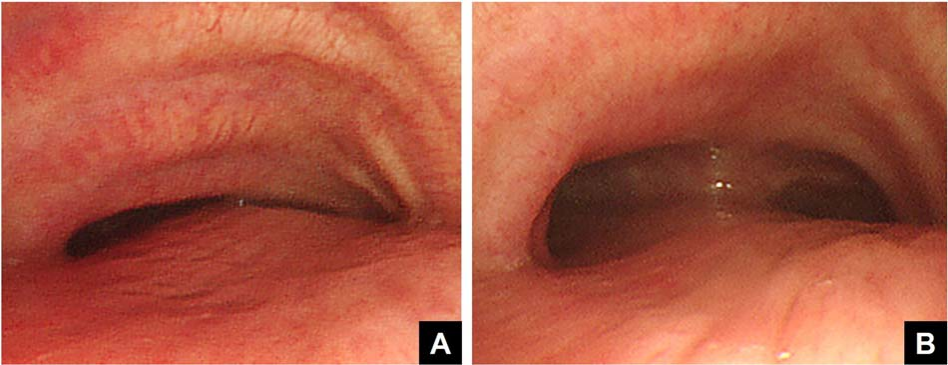
Pre- (A) and postoperative (B) bronchoscopic findings. Preoperatively, the right main bronchus is severely stenotic (A). The stenosis of the right main bronchus improves after performing the Nuss procedure (B).

Based on these imaging tests, surgery for oropharyngeal cancer caused breathing difficulties due to a leftward shift of the trachea. The sternum’s depression led to severe narrowing of the right main bronchus, and age-related factors added to the respiratory distress. We performed a Nuss procedure for PE repair to release the stenosis of the right main bronchus surgically. As thoracoscopy revealed a depressed sternum, we corrected the sternal depression by sternal elevation using two pectus bars. Postoperative chest CT showed improvement in the sternal depression (HI:4.6, Fig. 3B) and right main bronchial stenosis (Fig. 4B). Furthermore, shortness of breath was relieved postoperatively.

## Discussion

Surgical indications for PE include cardiopulmonary dysfunction and cosmetic issues [2, 3]. Although there are several reports on respiratory dysfunction caused by PE [2], only a few reports indicate airway stenosis associated with physical compression. In the present case, two factors were considered to cause the onset of dyspnea. The first is the leftward deviation of the trachea that occurs after surgery for oropharyngeal cancer. The second is the decline in thoracic retention with age. Pneumonectomy can cause mediastinal deviation, leading to a rare condition called postpneumonectomy syndrome [4]. A recent case report suggests that mediastinal dissection during laryngopharyngeal surgery may decrease mediastinum support causing it to shift, which, in turn, may narrow the right main bronchus due to tracheal shifting.

Reports indicate that PE worsens with age [5]. Nagasao *et al.* reported that the anterior abnormal part of the chest wall muscles gradually sags under the influence of the negative thoracic pressure [6]. This condition is often observed in patients with Poland syndrome [7]. After surgery for oropharyngeal cancer, the thoracic muscle strength may have weakened due to aging and malnutrition, further aggravating the PE and respiratory distress.

Based on these findings, we used the Nuss method to relieve respiratory distress caused by stenosis of the right main bronchus. Stent treatment using bronchoscopy was not effective due to bone compression, so we used strong thoracic elevation with a pectus bar [8]. Postoperative chest CT showed improvement in the sternal depression and right main bronchial stenosis. Furthermore, shortness of breath was relieved postoperatively.

In conclusion, we performed the Nuss procedure to treat PE with dyspnea after surgery for oropharyngeal cancer. The mechanism responsible for the development of airway obstruction in this patient may be important and deserves discussion.

## Conflict of interest statement

None declared.

## Funding

This work was supported by JSPS KAKENHI (Grant Number 21K08627).
